# Supplementary material for: Computational Modeling of the Photon Transport, Tissue Heating, and Cytochrome C Oxidase Absorption during Transcranial Near-Infrared Stimulation
Source: Brain Sci. 2019 Jul 27;9(8):179. doi: 10.3390/brainsci9080179 (PMC6721367; doi:10.3390/brainsci9080179)
Supplement: Supplementary file 1 [file brainsci-09-00179-s001.zip › brainsci-544481-suppl/Supplementary Material.pdf]

Supplementary Materials:

1. Calculation of Absorption Coefficient

Absorption Coefficient, μ_a, of a tissue as a whole is the sum of the absorption coefficient of all the chromophores in a tissue[1]. The absorption coefficient of the whole tissue is given as:

$$\mu_a = ln(10) \sum_i C_i \varepsilon_i \tag{1}$$

where C_i is the concentration of the chromophore i and ε_i is the specific extinction coefficient of the chromophore i. If the chromophores do not have the molecular concentration specified, then equation 1 can be rewritten in terms of volume fraction as:

$$\mu_a = \sum_i f_{v,i} \mu_{a,i} \tag{2}$$

where $f_{v,i}$ is the volume fraction of the chromophore i in the tissue and $\mu_{a,i}$ is the absorption coefficient of the pure chromophore component i.

Thus, we can calculate the absorption contribution of each chromophore by two methods:

1. Specific extinction coefficient of chromophore and concentration of chromophore in the tissue
2. Volume fraction of the chromophore and the absorption coefficient of the pure chromophore

1.1. Water

The absorption coefficient of pure water in biological tissues is given as:

**Component**	**Absorption Coefficient** $(1/m)$		
	**630nm**	**700nm**	**810nm**
Water	0.33	0.62	2.07

1.1.1. Gray Matter

The volume fraction of water in human brain is 80%. Thus, by equation 2, the absorption coefficient in gray matter due to the contribution of water is:

**Component**	**Absorption Coefficient** $(1/m)$		
	**630nm**	**700nm**	**810nm**
Water Contribution	0.264	0.5	1.66

1.1.2. White Matter

The volume fraction of water in human brain is 70%. Thus, by equation 2, the absorption coefficient in white matter due to the contribution of water is:

**Component**	**Absorption Coefficient** $(1/m)$		
	**630nm**	**700nm**	**810nm**
Water Contribution	0.23	0.43	1.45

1.2. Fat

The absorption coefficient of fat in biological tissues is given as:

Component	Absorption Coefficient $(1/m)$		
	**630nm**	**700nm**	**810nm**
Fat	0.43	0.32	0.53

1.2.1. Gray Matter

The fraction of fat in dry gray matter is 46% where the dry gray matter is 100 - %water of the gray matter. Thus, the volume fraction of fat in the gray matter is 20x46/100=9.2% and from equation 2, the absorption coefficient in gray matter due to the contribution of fat is:

Component	Absorption Coefficient $(1/m)$		
	**630nm**	**700nm**	**810nm**
Fat Contribution	0.04	0.03	0.05

1.2.2. White Matter

The fraction of fat in dry white matter is 64.6% where the dry white matter is 100 - %water of the white matter. Thus, the volume fraction of fat in the white matter is 30x64.6/100=19.4% and from equation 2, the absorption coefficient in white matter due to the contribution of water is:

Component	Absorption Coefficient $(1/m)$		
	**630nm**	**700nm**	**810nm**
Fat Contribution	0.08	0.06	0.1

1.3. Oxyhemoglobin

The absorption coefficient of oxyhemoglobin in 100% blood is[2]:

Component	Absorption Coefficient $(1/m)$		
	**630nm**	**700nm**	**810nm**
Oxyhemoglobin	520	450	830

1.3.1. Gray Matter

The blood volume fraction in gray matter, B is 5.2%[3].
The hemoglobin oxygen saturation in gray matter, S is 52%[4].
The absorption contribution of oxyhemoglobin in tissue is given by[1]:

$$\mu_a = BS\mu_{a,oxy} \tag{3}$$

Thus, absorption contribution of oxyhemoglobin in gray matter at the three wavelengths are:

Component	Absorption Coefficient $(1/m)$		
	**630nm**	**700nm**	**810nm**
Oxyhemoglobin Contribution	14.06	12.17	22.44

1.3.2. White Matter

The blood volume fraction in white matter, B is 2.7%[3].
The hemoglobin oxygen saturation in the white matter, S is 24%[4].

The absorption contribution of oxyhemoglobin in tissue is given by[1]:

$$\mu_a = BS\mu_{a,oxy} \tag{4}$$

Thus, absorption contribution of oxyhemoglobin in white matter at the three wavelengths are:

**Component**	**Absorption Coefficient** $(1/m)$		
	**630nm**	**700nm**	**810nm**
Oxyhemoglobin Contribution	3.37	2.92	5.38

1.4. Dexyhemoglobin

The absorption coefficient of deoxyhemoglobin in 100% blood is[2]:

**Component**	**Absorption Coefficient** $(1/m)$		
	**630nm**	**700nm**	**810nm**
Deoxyhemoglobin	2920	1370	620

1.4.1. Gray Matter

The blood volume fraction in gray matter, B is 5.2%[3].

The hemoglobin oxygen saturation in gray matter, S is 52%[4].

The absorption contribution of deoxyhemoglobin in tissue is given by[1]:

$$\mu_a = B(1-S)\mu_{a,deoxy} \tag{5}$$

Thus, absorption contribution of deoxyhemoglobin in gray matter at the three wavelengths are:

**Component**	**Absorption Coefficient** $(1/m)$		
	**630nm**	**700nm**	**810nm**
Deoxyhemoglobin Contribution	73	34.2	15.48

1.4.2. White Matter

The blood volume fraction in white matter, B is 2.7%[3].

The hemoglobin oxygen saturation in the white matter, S is 24%[4].

The absorption contribution of deoxyhemoglobin in tissue is given by[1]:

$$\mu_a = B(1-S)\mu_{a,deoxy} \tag{6}$$

Thus, absorption contribution of deoxyhemoglobin in white matter at the three wavelengths are:

**Component**	**Absorption Coefficient** $(1/m)$		
	**630nm**	**700nm**	**810nm**
Deoxyhemoglobin Contribution	59.92	28.11	12.72

1.5. Reduced Cytochrome c Oxidase

The absorption coefficient of reduced cytochrome c oxidase per 1 molar concentration of the chromophore is:

Component	Absorption Coefficient/mole $(1/m/M)$		
	**630nm**	**700nm**	**810nm**
Reduced Cytochrome c Oxidase	1190	540	350

1.5.1. Gray Matter

The concentration of reduced cytochrome c oxidase is $3.4\mu M$[5]. Thus, by eqn. 1, the absorption contribution of reduced cytochrome c oxidase in gray matter is:

Component	Absorption Coefficient $(1/m)$		
	**630nm**	**700nm**	**810nm**
Reduced Cytochrome c Oxidase Contribution	4.25	1.97	1.26

1.5.2. White Matter

The concentration of reduced cytochrome c oxidase is $0.1\mu M$[5]. Thus, by eqn. 1, the absorption contribution of reduced cytochrome c oxidase in white matter is:

Component	Absorption Coefficient $(1/m)$		
	**630nm**	**700nm**	**810nm**
Reduced Cytochrome c Oxidase Contribution	0.13	0.06	0.04

1.6. Oxidized Cytochrome c Oxidase

The absorption coefficient of oxidized cytochrome c oxidase per 1 molar concentration of the chromophore is:

Component	Absorption Coefficient/mole $(1/m/M)$		
	**630nm**	**700nm**	**810nm**
Oxidized Cytochrome c Oxidase	1950	760	900

1.6.1. Gray Matter

The concentration of oxidized cytochrome c oxidase is $18\mu M$. Thus, by eqn. 1, the absorption contribution of oxidized cytochrome c oxidase in gray matter is:

Component	Absorption Coefficient $(1/m)$		
	**630nm**	**700nm**	**810nm**
Oxidized Cytochrome c Oxidase Contribution	35.64	14.04	16.2

1.6.2. White Matter

The concentration of reduced cytochrome c oxidase is $0.1\mu M$. Thus, by eqn. 1, the absorption contribution of reduced cytochrome c oxidase in white matter is:

Component	Absorption Coefficient $(1/m)$		
	**630nm**	**700nm**	**810nm**
Oxidized Cytochrome c Oxidase Contribution	2.38	0.94	1.08

1.7. Whole Tissue Absorption Coefficient

The whole tissue absorption coefficient is the sum of the absorption coefficients of all individual components given by(adapted from [1]):

$$\mu_a = BS\mu_{a,oxy} + B(1-S)\mu_{a,deoxy} + W\mu_{a,water} + F\mu_{a,fat} + M\mu_{a,chromophore} \quad (7)$$

where B is the blood volume fraction, S is oxygen saturation, W is water volume fraction, F is fat volume fraction, M is molar concentration.

1.7.1. Gray Matter

Component	Absorption Coefficient $(1/m)$		
	**630nm**	**700nm**	**810nm**
Whole Tissue Gray Matter	127.25	62.91	57.09

1.7.2. White Matter

Component	Absorption Coefficient $(1/m)$		
	**630nm**	**700nm**	**810nm**
Whole Tissue White Matter	66.11	32.52	20.77

2. Importance of Cytochrome c Oxidase

The calculated values show a considerable absorption is contributed by the cytochrome c oxidase; especially it's oxidized state. The consideration of Cytochrome c oxidase is due to its role in tissue metabolism, especially in aerobic metabolism of glucose. The oxidation of NADH, a significant part of oxidative phosphorylation involves the oxidation of electron transport protein, Cytochrome C by the CCO(electron acceptor) after the successive acceptance of electron from NADH to produce ubiquinone and oxidation of ubiquinone at complex I and III respectively. The electrons are ultimately utilized to reduce oxygen to water. Complex IV contributes mostly in the proton electrochemical potential, thereby driving mitochondrial ATP. We have considered an average estimate of oxidized CCO concentration in the gray and white matter[5]. The binuclear copper center(CuA) of CCO is what dominates the NIR-dependency of the CCO[6].
It is to be also noted that the NIR signal absorption can also be affected by other cytochrome redox centers and oxygen intermediates which have not been considered in the study.

3. Offset of Tissue Fat and Water

In the tissue, fat, and water offset each other with fat volume fraction [0 by 0.1 to 0.7] as water volume fraction [0.7 by 0.1 to 0.1], so that combined fat and water volume fraction is 0.7[1].

References

1. Jacques, S.L. Optical properties of biological tissues: a review. *Physics in Medicine & Biology* **2013**, *58*, R37.
2. Prahl, S. Tabulated molar extinction coefficient for hemoglobin in water. *http://omlc. ogi. edu/spectra/hemoglobin/summary. html* **1999**.
3. Leenders, K.; Perani, D.; Lammertsma, A.; Heather, J.; Buckingham, P.; Jones, T.; Healy, M.; Gibbs, J.; Wise, R.; Hatazawa, J.; others. Cerebral blood flow, blood volume and oxygen utilization: normal values and effect of age. *Brain* **1990**, *113*, 27–47.
4. Rejmstad, P.; Zsigmond, P.; Wårdell, K. Oxygen saturation estimation in brain tissue using diffuse reflectance spectroscopy along stereotactic trajectories. *Optics express* **2017**, *25*, 8192–8201.
5. Johansson, J.D.; Wårdell, K. Intracerebral quantitative chromophore estimation from reflectance spectra captured during deep brain stimulation implantation. *Journal of biophotonics* **2013**, *6*, 435–445.
6. Bale, G.; Elwell, C.E.; Tachtsidis, I. From Jöbsis to the present day: a review of clinical near-infrared spectroscopy measurements of cerebral cytochrome-c-oxidase. *Journal of biomedical optics* **2016**, *21*, 091307.
