# Supplementary figures and images for: Computational Modeling of the Photon Transport, Tissue Heating, and Cytochrome C Oxidase Absorption during Transcranial Near-Infrared Stimulation
Source: Brain Sci. 2019 Jul 27;9(8):179. doi: 10.3390/brainsci9080179 (PMC6721367; doi:10.3390/brainsci9080179)

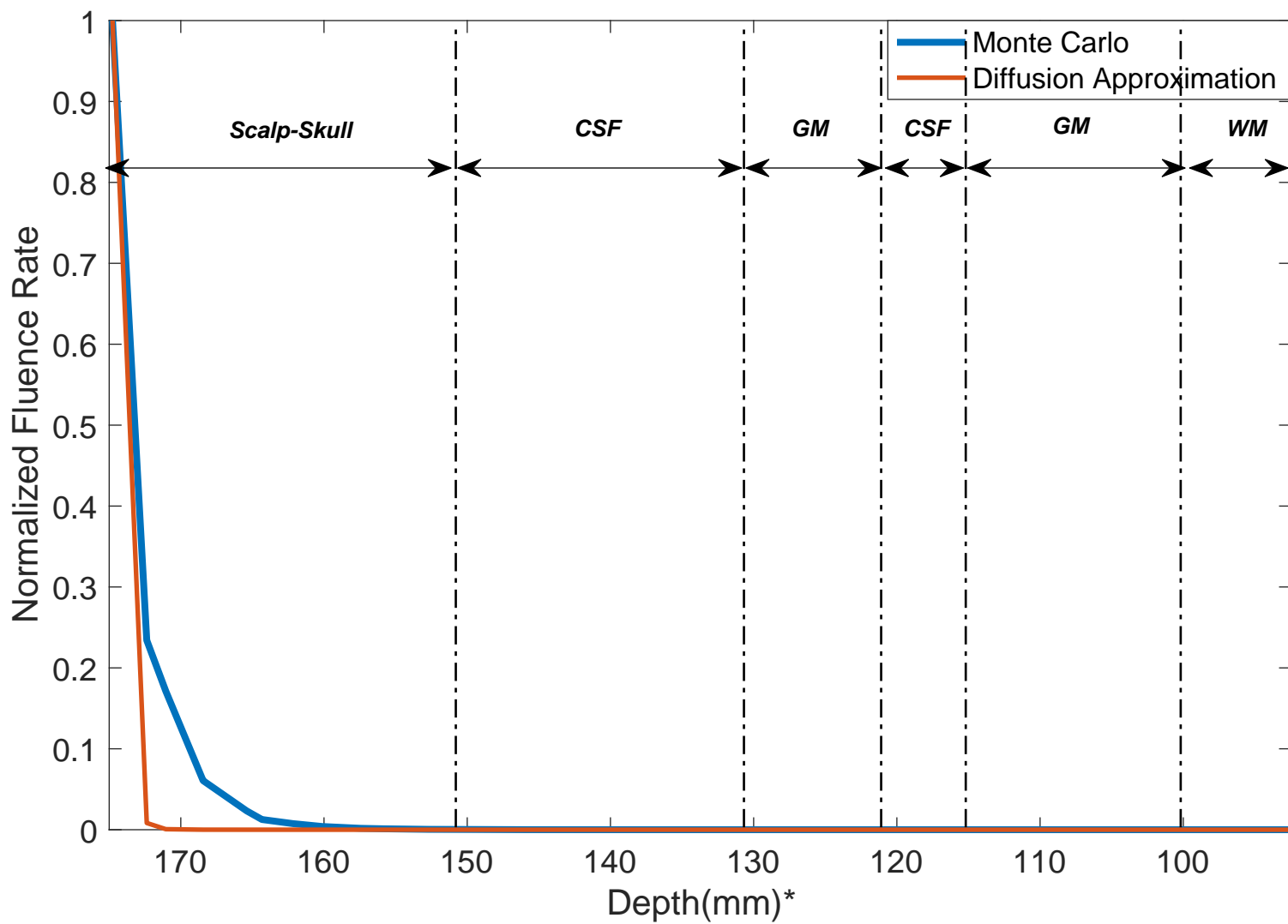

Supplement: Supplementary file 1 [file brainsci-09-00179-s001.zip › brainsci-544481-suppl/Figure S1 Monte Carlo vs Diffusion Approximation.pdf]
